# Mediterranean Diet Adherence in a Sample of Italian Adolescents Attending Secondary School—The “#facciamoComunicAzione” Project

**DOI:** 10.3390/nu13082806

**Published:** 2021-08-16

**Authors:** Monica Dinu, Sofia Lotti, Giuditta Pagliai, Livia Pisciotta, Mariacarla Zavatarelli, Matilde Borriello, Roberto Solinas, Roberto Galuffo, Alessandro Clavarino, Ettore Acerra, Francesco Sofi, on behalf of the Working Group of the “#facciamoComunicAzione” Project

**Affiliations:** 1 Department of Experimental and Clinical Medicine, University of Florence, 50134 Florence, Italy; sofia.lotti@unifi.it (S.L.); giuditta.pagliai@gmail.com (G.P.); francesco.sofi@unifi.it (F.S.); 2 Department of Internal Medicine, University of Genoa, 16126 Genoa, Italy; livia.pisciotta@unige.it; 3 Istituto Professionale per l’Enogastronomia e l’Ospitalità Alberghiera “Marco Polo”, 16147 Genoa, Italy; prof.zavatarelli.mariacarla@marcopologenova.net (M.Z.); matilde.borriello@unige.it (M.B.); roberto.solinas@ipsiaodero.it (R.S.); 4 Educazione alla Salute, Ufficio Scolastico Regionale per la Liguria, 16121 Genoa, Italy; robertogaluffo@gmail.com; 5 Ordinamenti Scolastici, Politiche Formative, Diritto allo Studio, Comunicazione, Ufficio Scolastico Regionale per la Liguria, 16121 Genoa, Italy; clavarinoalessandro@gmail.com; 6 Direzione Generale, Ufficio Scolastico Regionale per la Liguria, 16121 Genoa, Italy; ettore.acerra1@istruzione.it

**Keywords:** Mediterranean diet, adherence, Medi-Lite, students

## Abstract

Our aim was to assess adherence to the Mediterranean diet in a group of 726 secondary school students (336 girls, 390 boys) who completed the web-based Medi-Lite questionnaire simultaneously, during school hours, at the “Istituto Professionale per l’Enogastronomia e l’Ospitalità Alberghiera Marco Polo” in Genoa, Italy. The mean adherence score was 9.28 ± 2.29, with significantly (*p* = 0.017) higher values in girls (9.5 ± 2.2) than boys (9.1 ± 2.4). As to the individual food components of the Medi-Lite score, 84% of students reported non-optimal consumption (i.e., the choice that yielded ≤ 1 point) of meat and meat products, and over 50% reported non-optimal consumption of vegetables, legumes, dairy products, and fish. Significant differences between girls and boys were observed for fruit (*p* = 0.003), cereals (*p* < 0.001), meat and meat products (*p* < 0.001), and dairy products (*p* = 0.003). By conducting a principal component analysis, we observed that Medi-Lite items on the consumption of some animal products (meat and meat products and dairy products) and some plant products (fruit, vegetables, and legumes) generated contrasting patterns of responses, denoting excessive consumption in the first case and underconsumption in the second. This result suggests the need for effective actions to promote healthy eating habits in young people.

## 1. Introduction

The Mediterranean diet is one of the most studied and appreciated dietary patterns in the scientific community [1]. It is characterized by a high consumption of plant foods, moderate consumption of fish, dairy products, and eggs, low consumption of meat and meat products, and olive oil as the main source of fat. Added to the UNESCO Intangible Cultural Heritage list in 2010, the Mediterranean diet has not only nutritional value, but also cultural and social value, and is presented as a model of a sustainable diet for health and the environment [2].

Despite the health benefits of the Mediterranean diet, widely documented in both epidemiological and clinical studies [1], current surveys indicate that many Mediterranean countries are moving away from this dietary pattern [3,4]. A process of dietary westernization has also emerged in the Italian population, particularly among younger generations [5,6,7]. In a recent large, nationally representative sample of Italian adolescents, more than half of the respondents reported not eating fruit and/or vegetables daily and more than 10% reported drinking carbonated, high-sugar drinks at least once a day [8]. These data are worrisome because unhealthy eating habits contribute to the development of overweight, obesity, and diet-related non-communicable diseases even at a young age, with serious implications for public health [3]. To encourage healthy food choices and promote innovative policies and targeted actions among young people, knowledge of their eating habits is essential. This knowledge can be obtained through surveillance and monitoring initiatives [9]. In previous studies, the integration of technology-based tools and active learning programs, such as Flash Mob, a group of people who gather in a public place to perform for a short period of time an event, have been shown to be effective in improving student engagement [10,11]. In the present study, we conducted a Flash Mob on the Mediterranean diet in a secondary school with the aim of evaluating adherence to the Mediterranean diet in students who completed the web version of the Medi-Lite questionnaire, an evidence-based tool to measure adherence to the Mediterranean diet [12].

## 2. Materials and Methods

### 2.1. Study Sample

A Flash Mob on the Mediterranean diet was held at the “Istituto Professionale per l’Enogastronomia e l’Ospitalità Alberghiera Marco Polo” in Genoa, Italy, on 3–4 March 2021. The Flash Mob was carried out by the 5th classes at the end of an educational and nutritional program that saw as protagonists the teachers of the classes and the students themselves (the “#facciamoComunicAzione” project). The teachers, together with the principal, presented the project to the students and their families and, after obtaining the approval of the school board, sent a letter to the families explaining the project and obtaining their consent to have their children participate in the initiative. No inclusion and/or exclusion criteria were established. 

### 2.2. The Web-Based Medi-Lite Score

The web-based version of the Medi-Lite score was developed in 2018 by NUME PLUS srl (Florence, Italy) and published online as a free version at http://www.medi-lite.com (accessed on 30 June 2021) after applying the copyright of the University of Florence, Italy. Compared to the paper version, published in its original version in 2014 [12], and validated in 2017 [13], the web-based version has undergone some changes, as previously described [14]. In particular, the 9 items of the original score have been divided into 20 different questions assessing the consumption, in terms of daily and/or weekly amounts, of 9 food groups: fruits, vegetables, legumes, cereals, meat and meat products, fish, dairy products, alcohol, and olive oil. For typical products of the Mediterranean diet (fruit, vegetables, cereals, legumes, and fish), 2 points are assigned to the highest category of consumption, 1 point to the intermediate category, and 0 to the lowest category. On the other hand, foods that are not typical of the Mediterranean diet (meat and meat products, dairy products) are assigned 2 points for the lowest category of consumption, 1 point for the intermediate category, and 0 for the highest category. Finally, 2 points are awarded for regular use of olive oil, 1 for frequent use, and 0 for occasional use. To improve the feasibility of the questionnaire for the general population, each food group is accompanied by a photographic database. Respondents are asked to indicate both their portion size (by selecting one of three options) and their frequency of consumption (by selecting an option from 0 to 6 or more times per day/week). Once completed, the web-based questionnaire calculates the sum of the scores assigned to each question and provides personalized guidance on how to improve the diet and increase adherence to the Mediterranean diet.

In this study, due to the age of the students, the question on alcohol consumption was not administered. Therefore, the final score ranged from 0 to 16 points and indicated low or high adherence to the Mediterranean diet. Suboptimal consumption for each food group composing the Medi-Lite score was defined as the choice that produced ≤ 1 point and corresponded to the following consumption levels: fruit < 2 portions/day, vegetables < 2.5 portions/day, legumes < 2 portions/week, cereals < 1.5 portions/day, meat and meat products > 1 portion/day, fish < 2.5 portions/week, dairy products > 1 portion/day, and non-regular use of olive oil. Both portion sizes, which differed across food categories, and consumption frequencies were defined based on data obtained in the analysis of cohort studies that estimated an association between the Mediterranean diet and overall mortality, as previously described [12].

### 2.3. Data Collection

All students were allowed to self-administer the questionnaire at the same time, during school hours, without any parent or teacher supervision. Before measuring adherence to the Mediterranean diet, they were asked to provide their gender. The response format was closed and structured. To preserve student privacy, responses were collected at the website http://www.medi-lite.com (accessed on 30 June 2021), ensuring that participants’ responses were protected. The IP address of the client computer was used to identify potential duplicate responses from the same user. In the event of duplicate responses, the most recent response was kept for analysis. Because all responses were anonymous and voluntary, ethics committee approval was not required.

### 2.4. Statistical Analysis

Statistical analysis was performed using the statistical package PASW 27.0 for Macintosh (SPSS Inc., Chicago, IL, USA). Descriptive statistics were used to analyze and report data. Histograms and boxplots were used to assess the distributional assumptions and to check for possible outliers. The results were expressed as mean ± standard deviation (SD), median and min–max range, or number and percentage (%), as appropriate. Differences between groups were estimated using the unpaired t-Student test. The Pearson’s chi-square was used to test for proportions. To understand whether the Medi-Lite items generated similar patterns of responses, a principal components analysis (PCA) with varimax rotation was performed. For all tests, a *p*-value < 0.05 was considered statistically significant.

## 3. Results

A total of 726 students aged 17–19 years participated in the Flash Mob and completed the web-based Medi-Lite questionnaire. Of these, 336 were girls (46.3%) and 390 were boys (53.7%). The scores obtained from the Medi-Lite questionnaire ranged from 2 to 15, with a median of 10. The mean adherence score was 9.28 ± 2.29, with significantly (*p* = 0.017) higher values in girls (9.50 ± 2.19) than boys (9.09 ± 2.35). Categorizing the Medi-Lite score into tertiles, we obtained three adherence groups: low (score ≤ 7; *n* = 162), moderate (score 8–10; *n* = 336), and high (score ≥ 11; *n* = 228). The prevalence rate of low adherents was significantly (*p* = 0.012) higher in boys (*n* = 101; 26%) than girls (*n* = 61; 18%). 

Regarding the individual food components of the Medi-Lite score, the meat and meat products group showed the highest number of students (*n* = 617; 84%) reporting suboptimal consumption (i.e., the choice that yielded ≤1 point). In addition, 319 (42%) students reported suboptimal consumption of fruit (<2 portions/day), 385 (53%) reported suboptimal consumption of vegetables (<2.5 portions/day), 474 (65%) reported suboptimal consumption of legumes (<2 portions/week), and 433 (61%) reported suboptimal consumption of fish (<2.5 portions/week). Surprisingly, 391 students (56%) reported not using extra virgin olive oil regularly. Cereal consumption, on the other hand, was in line with the principles of the Mediterranean diet in most of the sample (*n* = 571; 79%). 

**Figure 1:**
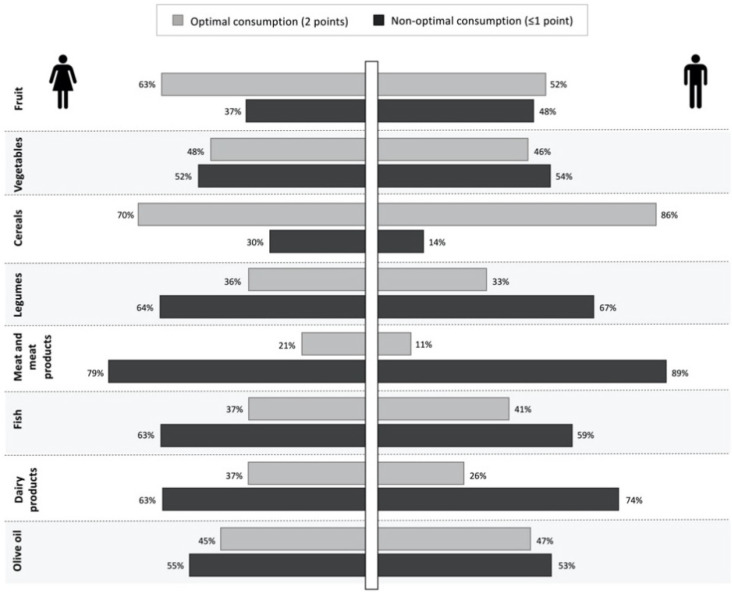
Percentages of girls and boys reporting optimal (2 points) and non-optimal (≤1 points) consumption of the individual food groups of the Medi-Lite score.

Significant differences between girls and boys, in terms of suboptimal consumption of individual food components of the Medi-Lite score, were observed for fruit (*p* = 0.003), cereals (*p* < 0.001), meat and meat products (*p* < 0.001), and dairy products (*p* = 0.003). As shown in Figure 1, for all these food groups except cereals, a greater proportion of boys than girls scored ≤1 point, denoting either overconsumption (i.e., for meat and meat products) or underconsumption (i.e., for fruit). 

**Figure 2:**
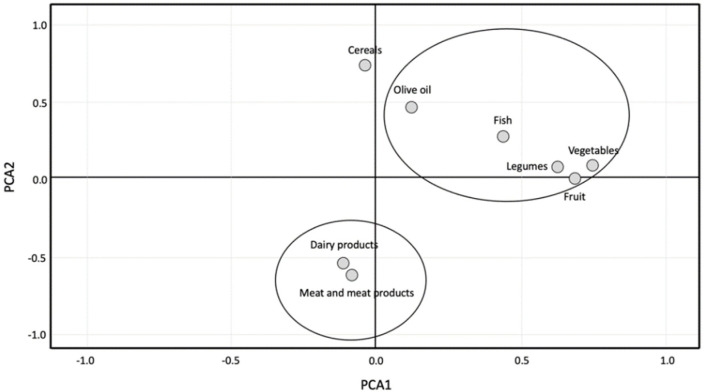
Principal component analysis of the individual food groups of the Medi-Lite score.

To understand the different patterns of consumption among the adolescents investigated, we conducted a PCA analysis of the Medi-Lite scores regarding the individual food components. As shown in Figure 2, the sample investigated was clustered into two main groups regarding the consumption of each specific food component of the score. 

A cluster of overconsumptions was identified for animal-based food products such as meat and meat products as well as dairy products. Another cluster was composed of fruit, vegetables, legumes, fish, and olive oil, which showed the same pattern of suboptimal consumption. 

## 4. Discussion

In the present study, we assessed adherence to the Mediterranean diet through the web-based Medi-Lite questionnaire in a group of secondary school students during a Flash Mob on the Mediterranean diet. The data obtained showed a moderate adherence to the Mediterranean diet, with high consumption of animal-based food products and low consumption of vegetables, legumes, fish, and olive oil. 

Nutrition is crucial for the development and long-term health of adolescents. Adopting good eating habits at a young age can protect against numerous health problems, such as obesity and cardiovascular disease [15]. In this context, the Mediterranean diet has been widely recognized as a healthy and sustainable dietary pattern. Consistent research has shown that greater adherence to this dietary pattern is linked to a lower risk of developing overweight and obesity during childhood [16,17] and increased lifespan and healthy ageing in adults [1]. Despite these benefits, Mediterranean populations are gradually moving away from this dietary pattern [4,18]. The causes are multiple and complex and are related to economic, cultural, and social constraints. The increased variety and availability of food and energy supplies, the urbanization of life, improved services and networks that can also lead to a more stressful lifestyle, and reduced time devoted to cooking are among the main drivers of this change, especially among young people [19]. Moreover, the increase in tourism and migration observed in recent decades has increased cultural and lifestyle exchanges between populations, including in terms of dietary habits [20].

In our study, the median value of adherence was 10 points on a scale of 0 to 16, denoting a moderate level of adherence to the Mediterranean diet in the population examined. These results are in line with previous studies reporting a low-to-moderate level of adherence to the Mediterranean diet among young populations living in the Mediterranean area. In a representative sample of Greek adolescents, only 8.3% of adolescents reported eating habits that followed the principles of the Mediterranean diet [21], and less than 50% of a sample of 3850 Spanish adolescents had an optimal Mediterranean diet [22]. In Italy, results of the Calabrian Sierras Community Study (CSCS), which investigated a population attending primary and secondary schools in a 14-city southern community, showed poor adherence to the Mediterranean diet in 18.4% of the children and adolescents analyzed [23]. Similar results were observed in Central Italy, in a survey of 1127 adolescents in Tuscany [24], and in Northern Italy, in five schools in the province of Novara, where adherence to the Mediterranean diet was poor in 16.7% of students [25].

Our data also showed that boys were more likely than girls to show low adherence to the Mediterranean diet, which is consistent with previous studies conducted in Greek and Balearic Islands adolescents [21,26]. However, the relationship between gender and adherence to the Mediterranean diet in this age group has not always been confirmed. A systematic review of cross-sectional studies conducted on Spanish, Greek, and Italian adolescents did not find an unequivocal relationship between low adherence to the Mediterranean diet and gender [5]. Considering all available studies on the topic, the meta-analysis by Iaccarino Idelson et al. also concluded that the evidence in children and adolescents is conflicting and does not support differences in adherence to the Mediterranean diet by gender [6].

Finally, the consumption of the individual food components of the Medi-Lite score reported by our group of students was similar to that reported by previous studies examining the dietary habits of adolescents living in countries bordering the Mediterranean Sea [7,27,28]. In fact, almost 50% of students consumed insufficient amounts of fruit and vegetables, less than 60% ate legumes and fish regularly, and more than 80% consumed meat and meat products more than once a day. Similar data were recently reported across EU countries in the latest Health at Glance Report 2020 [29] and in the Eating Study as a KiGGS Module (EsKiMo) II, conducted on 1353 adolescents aged 15–17 years [30]. Notably, only one in nine adolescents reported eating five or more servings of fruit and vegetables per day, while meat consumption was well above recommendations. Because eating habits established in adolescence tend to be maintained into adulthood, increasing the consumption of fruit, vegetables, fish, and legumes among adolescents, and reducing the intake of meat and meat products, is an important public health concern. 

The study has some limitations that should be discussed. First, the information collected was self-reported. This may have led to recall bias and misreporting due to the nature of the study and the young age of the participants. Second, the study sample was from a single institution and was not representative of the population of students attending secondary schools. In addition, adherence to the Mediterranean diet was assessed by considering the daily and weekly intake of eight food groups composing the Medi-Lite score: fruit, vegetables, cereals, legumes, meat and meat products, fish, dairy products, and olive oil. As previously described [12], both portion sizes and consumption frequencies were defined based on data obtained in the analysis of cohort studies that estimated the association between the Mediterranean diet and overall mortality. We are aware that this approach may be questionable, as are all other approaches, but, to date, there is no gold-standard method for defining adherence to the Mediterranean diet. Second, our sample consisted of a group of students for whom we did not have specific information about cultural background, access to food, or other factors such as physical activity and smoking habits, all of which are important predictors of dietary habits. This may have influenced, for example, the low consumption of olive oil reported by the students surveyed. To confirm these data, similar initiatives in the future will need to collect more information on the lifestyle and sociodemographic characteristics of the participants. Despite these limitations, this study also had some strengths, including the relatively large sample size, the use of a validated questionnaire specifically designed to measure adherence to the Mediterranean diet at an individual level, and the novel communication approach. In previous studies, the use of Flash Mobs as a teaching tool has been shown to be effective in increasing student enthusiasm and interest in specific initiatives [11], as well as being useful for collecting research data [31].

## 5. Conclusions

In conclusion, the data obtained in a group of secondary school students aged 17–19 years showed a moderate adherence to the Mediterranean diet, emphasizing the need to plan educational interventions aimed at improving the behaviors of adolescents. In particular, the consumption of fruit, vegetables, legumes, and fish should be encouraged, as well as the reduced consumption of meat and meat products. 

## Data Availability

Data will be made available on request.
